# Platelet Reduction after Aortic Bioprosthesis Implantation: Results from the PORTRAIT Study

**DOI:** 10.3390/jcm12237414

**Published:** 2023-11-29

**Authors:** Federica Jiritano, Giuseppe Filiberto Serraino, Michele Di Mauro, Massimo Borelli, Roberto Scrofani, Leonardo Patanè, Elena Caporali, Matteo Matteucci, Dario Fina, Mariusz Kowalewski, Francesco Pollari, Theodor Fischlein, Giuseppe Visicchio, Domenico Paparella, Giosuè Falcetta, Andrea Colli, Pasquale Mastroroberto, Giangiuseppe Cappabianca, Roberto Lorusso

**Affiliations:** 1 Cardiac Surgery Unit, Department of Experimental and Clinical Medicine, University “Magna Graecia” of Catanzaro, Viale Europa, 88100 Catanzaro, Italy; serraino@unicz.it (G.F.S.); mastroroberto@unicz.it (P.M.); 2 Cardio-Thoracic Surgery Department, Heart & Vascular Centre, Maastricht University Medical Centre (MUMC), Cardiovascular Research Institute Maastricht (CARIM), 6229 HX Maastricht, The Netherlands; mdimauro1973@gmail.com (M.D.M.); matteomatteucci87@gmail.com (M.M.); kowalewskimariusz@gazeta.pl (M.K.); robertolorussobs@gmail.com (R.L.); 3 Thoracic Research Centre, Collegium Medicum Nicolaus Copernicus University, Innovative Medical Forum, 85-094 Bydgoszcz, Poland; 4 UMG School of PhD Programmes Life Sciences & Technologies, University “Magna Graecia” of Catanzaro, 88100 Catanzaro, Italy; 5 Cardiac Surgery Unit, Luigi Sacco Hospital, 20157 Milan, Italy; roberto.scrofani@policlinico.mi.it; 6 Cardiology Cardiac Surgery Department (Centro Cuore), Centro Clinico Diagnostico G.B. Morgagni, 95125 Catania, Italy; lpatane@centrocuore.it; 7 Cardiac Surgery, Istituto Cardiocentro Ticino, 6900 Lugano, Switzerland; dottoressacaporali@gmail.com; 8 Department of Surgical and Morphological Sciences, Circolo Hospital, University of Insubria, 21100 Varese, Italy; giangi.cappabianca@googlemail.com; 9 Clinical Department of Cardiac Surgery, Central Clinical Hospital of the Ministry of Interior and Administration, Centre of Postgraduate Medical Education, 02-097 Warsaw, Poland; 10 Department for the Treatment and Study of Cardiothoracic Diseases and Cardiothoracic Transplantation, IRCCS-ISMETT, 90133 Palermo, Italy; 11 Klinikum Nürnberg, Cardiovascular Center, Paracelsus Medical University, 90419 Nuremberg, Germany; francesco.pollari@klinikum-nuernberg.de (F.P.); theodor.fischlein@klinikum-nuernberg.de (T.F.); 12 Department of Cardiac Surgery, Santa Maria Hospital, GVM Care & Research, 70124 Bari, Italy; visicchio@virgilio.it; 13 Dipartimento Scienze Mediche e Chirurgiche, Università di Foggia, 71122 Foggia, Italy; domenico.paparella@unifg.it; 14 University Hospital—Section of Cardiac Surgery, 56124 Pisa, Italy; giosuefalcetta@gmail.com (G.F.); colli.andrea.bcn@gmail.com (A.C.)

**Keywords:** platelet, biological prosthesis, aortic valve replacement, thrombocytopenia

## Abstract

**Background**: Platelet count reduction (PR) is a common but unclear phenomenon that occurs after aortic bioprosthesis valve implantation (bio-AVR). This study aimed to investigate the occurrence and clinical impact of PR in patients receiving stented, rapid deployment (RDV), or stentless bioprostheses. **Methods**: 1233 adult bio-AVR patients were enrolled. Platelet count variation, early post-operative adverse events, and in-hospital mortality were analysed. **Results**: 944 patients received a stented valve, an RDV was implanted in 218 patients, and 71 patients had a stentless bioprosthesis. In all groups, the platelet count at discharge was lower than the baseline values (*p* < 0.001). The percentage of PR was 27% in the stented group, 56% in the RDV group, and 55% in the stentless group. A higher platelet reduction, reaching the minimum platelet value, was observed in the RDV (mean: −30.84, standard error (SE): 5.91, *p* < 0.001) and stentless (mean: 22.54, SE: 9.10, *p* = 0.03) groups compared to the stented group. A greater PR occurred as the size of the bioprosthesis increased in RDV (*p* = 0.01), while platelet count variation was not directly proportional to the stented bioprosthesis size (*p* < 0.001). PR was not affected by cardiopulmonary bypass (mean: −0.00, SE: 0.001, *p* = 0.635) or cross-clamp (mean: −0.00, SE: 0.002, *p* = 0.051) times in any of the groups. RDV subjects experienced more in-hospital adverse events. PR was found to be associated with ischemic strokes in the overall population. **Conclusions**: Bio-AVR is associated with significant but transient PR. RDV patients more likely experience significant PR and related adverse clinical events. PR is associated with ischemic strokes, regardless of the bioprosthesis type.

## 1. Introduction

Recent years have seen a rise in interest and lively debate surrounding peri-operative platelet count decrease (PR) following aortic biological prosthesis implantation [1,2,3,4,5,6]. Several explanations for this phenomenon have been put up since patient-related risk factors were ruled out [1,2,3,4,5,6]. At first, it was thought that PR was caused by a particular biological device: a stentless valve [2,4]. This assumption, nevertheless, has been considered less accurate over time as other novel tissue valves, both surgical and transcatheter, have been linked to PR [1,7]. The discussion, consequently, has concentrated on biochemical and mechanical mechanisms that could lead to PR [1]. On the one hand, the blood’s interactions with the artificial valves may result in inflammation, alterations in the metabolic biochemistry and morphology of the platelets, and malfunctioning and lysis caused by the receptors [2,3,5,8]. Contrarily, mechanical platelet destruction brought on by shear stress through a prosthetic valve could result in platelet activation, aggregation, and the production of procoagulant microparticles, as well as platelet dysfunction, shedding of surface receptors, and, consequently, bleeding complications [2,5,6,9]. To date, however, there is still uncertainty concerning the relevance and clinical impact of this phenomenon after aortic biological valve replacement [1,2,6,9].

The purpose of the PORTRAIT (Post-Operative Thrombocytopenia After Bio-prosthesis Implantation) study is to look into changes in platelet count following the implantation of an aortic tissue valve. The purpose of the current sub-study is to specifically identify PR’s occurrence and clinical impact in three different categories of surgical bioprostheses.

## 2. Materials and Methods

### 2.1. Study Population

The present trial is a sub-study of the PORTRAIT study (Post-Operative Thrombocytopenia After Bio-prosthesis Implantation—trial registration: clinicaltrials.gov, NCT03835598)—a retrospective, multicenter, observational trial aimed at evaluating the occurrence of peri-operative thrombocytopenia and analysing the eventual clinical impact of the phenomenon. The study cohort consisted of adult patients who underwent isolated aortic valve replacement with a biological prosthesis between February 2011 and December 2019 in 9 different centres.

Patients were drawn from the PORTRAIT database. Patients were excluded in the following cases: (1) pre-operative platelet count was <100,000/uL; (2) an oncologic disease; (3) infection or inflammation disorder; (4) use of drugs (antibiotics, nonsteroidal anti-inflammatory drugs) inducing platelet count reduction (<3 months); and (5) recent percutaneous cardiac intervention (<1 month). This study was approved by the Institutional Review Board of the Maastricht University Medical Centre+ (Principal Investigators’ Centre, approval date: 23 January 2019, METC 2018-0923), and the need for individual patient consent was waived by the committee.

The study protocol was approved by each center’s local ethical committee and was carried out in accordance with the Declaration of Helsinki criteria for patient data usage and evaluation. To record important information, clinical histories, and examination data from medical records, a unified patient dataset was employed.

### 2.2. Study Endpoints

The primary endpoints of this study were: (1) the minimum platelet count during the hospital stay, the time until that minimum was reached, and the platelet count at discharge; (2) the rate of patients with a platelet count <100,000/uL; and (3) the effect of the prosthesis size, cardiopulmonary bypass, and cross-clamp times on platelet count variation.

The secondary endpoints were: (1) the need for transfusions of packed red blood cells (RBCs), platelets, or fresh-frozen plasma (FFP); (2) bleeding and re-thoracotomy events; (3) total blood-loss via drainages; (4) thrombotic and cardiovascular events; and (5) in-hospital mortality.

### 2.3. Definition

Platelet count reduction was defined according to the following formula:

$$PR=\left(\frac{Mean\;baseline\;platelet\;count-Mean\;lowest\;platelet\;count}{Mean\;baseline\;platelet\;count}\right)\times 100$$

### 2.4. Surgical and Post-Operative Details

According to the surgeon’s preference, a full median sternotomy, a mini-sternotomy, or a right thoracotomy was performed, and a cardiopulmonary bypass (CPB) was employed in every patient. The surgeon chose the prosthesis to implant. The platelet count was determined preoperatively, on the day of the surgery, and every day until Day 5 in the postoperative period.

### 2.5. Statistical Analysis

Continuous variables are expressed as the mean and standard deviation, or median and quartiles, respectively, for normally or non-normally distributed variables (as tested by the Shapiro–Wilk test) and were compared using Student’s *t*-test (or the Wilcoxon–Mann–Whitney U test, as appropriate); ANOVAs (followed by Tukey post hoc test) were used for multiple comparisons. Proportions are expressed as percentages and compared using the χ^2^ test or Fisher’s exact test, as appropriate. In order to analyse the blood product transfusions, a Poisson distributed generalised linear model was carried out. The mixed-model effect was determined to analyse the repeated measures of the platelet count. A LOESS (local regression) analysis was applied in order to assess the effect of CPB and X-Clamp time on platelet count variation. In order to improve the balance between the three different groups, a propensity score (PS) model was built. Then, a propensity score regression model was obtained by using the type of prosthesis as the target variable. Then, the resulting weights were computed using a formula that allows for the estimation of the treatment effects in the overlap population: 1-PS when the participant is from the target population; PS when the participant is from the original population. This method of PS weighting is defined as “overlap”; standardized mean differences below 0.20 were considered a good balance (Supplementary Figure S1) [10], and overlap weight was used to adjust the univariate and multivariate results. Differences were considered significant at *p* value < 0.05. Statistical data analysis was performed using JASP software v0.11.1 [11] and R Statistical Software v 4.3.2 [12]. For overlap PS weighting, the R package PS weight was used [13]. 

## 3. Results

A total of 1233 patients were included in the study; 944 patients received a stented bioprosthesis (Stented Group), 218 patients received a rapid deployment valve (RDV Group), and 71 patients had a Stentless bioprosthesis (Stentless Group). The pre-operative demographics of the patients are shown in Table 1. 

Patients in the Stented group were significantly younger (overall *p* = 0.022). The most commonly used surgical approach for Stented (62%) and Stentless (80%) tissue valves was a full sternotomy; a mini-sternotomy (44%) or a right thoracotomy (10%) were the most frequently used approaches for RDV patients (Supplementary Table S1). RDV implantation was associated with shorter CPB and cross-clamp times; stentless bioprosthesis implantation instead required longer times (Supplementary Table S1). However, after applying overall PS weighting, the three groups seemed to be more balanced, as differences for all the variables in Table 1 and Supplementary Table S2 (except for CPB and cross-clamp times) are below a standardized mean difference of 0.20 (Supplementary Figure S1). 

Patients were given either antiplatelet or anticoagulant drugs after the prosthesis was implanted. Specifically, low molecular weight heparin was administered until the patient could be mobilized. The patients were then given antiplatelet or anticoagulant medication based on their physician’s choice. Aspirin was used by 48% of stented patients, 54% of RDV patients, and 70% of stentless patients. Warfarin was given to 45% of stented bioprosthesis patients, 39% of RDV patients, and 26% of stentless bioprosthesis patients. Only 3.1% of stented patients and 3% of RDV individuals received dual antiplatelet treatment (DAPT). No DAPT was administered to stentless patients.

### 3.1. Primary Outcomes (Unweighted Analysis)

As per the inclusion criteria, the three groups had a pre-operative platelet count > 100,000/uL. No difference was found in the preoperative platelet count between groups (*p* = 0.700). In all groups, the platelet count at discharge was lower than the baseline values (*p* < 0.001). In detail, RDV showed a significantly greater PR than the stented bioprostheses at discharge (mean: −17.73, Standard Error (SE): 6.46, *p* = 0.01), while no significant differences were found between the other groups. The lowest platelet count reached by a patient occurred on Post-Operative Day (POD) 3 in all groups (Stented: 14 × 10^3^/uL; RDV: 6 × 10^3^/uL; Stentless: 25 × 10^3^/uL), while the minimum platelet count mean occurred between POD2 and 3 in all groups (Table 2).

The percentage of PR was 27% in the stented group, 56% in the RDV group, and 55% in the stentless group. The higher PR to minimum platelet value was observed in the RDV (mean:−30.84, SE:5.91, *p* < 0.001) and Stentless (mean: 22.54, SE: 9.10, *p* = 0.03) groups—both showing a similar PR (mean: −8.30; SE: 9.73, *p* = 0.66) compared to the stented group. Both RDV and Stentless patients showed the highest number of patients with a platelet count < 100,000/uL on POD2 and 3 (POD2: RDV = 54% vs. Stentless = 44% vs. Stented = 18%, *p* < 0.001; POD 3: RDV = 50% vs. Stentless = 55% vs. Stented = 14%, *p* < 0.001; Table 2).

**Figure 1:**
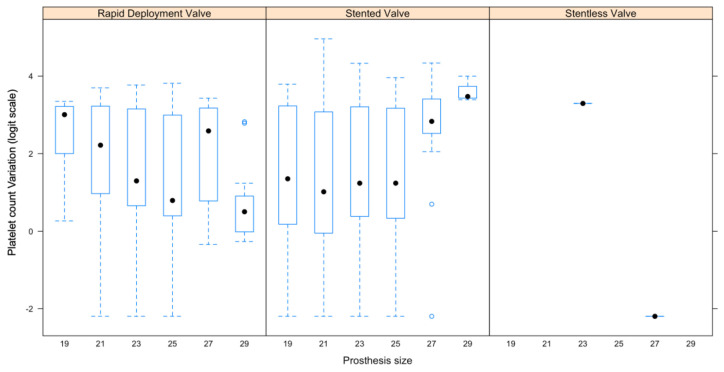
Correlation between platelet count variation and the biological prosthesis size.

Moreover, platelets changed according to the bioprosthesis size (Figure 1). Platelet count variation was calculated according to the following formula:

$$\left(\frac{Baseline\;platelet\;count-Discharge\;platelet\;count\;}{Baseline\;platelet\;count}\right)\times 100$$

After the application of a logit function, a greater platelet count drop was observed as the size of the bioprosthesis increased in the RDV group (mean:−0.13, SE: 0.05, *p* = 0.01). Although not statistically significant, a similar correlation was found for the stentless bioprostheses (mean: −1.23, SE: 0.67, *p* = 0.07). Conversely, platelet count variation in the stented group was not directly proportional to the valve size (mean: 0.23, SE: 0.06, *p* < 0.001).

**Figure 2:**
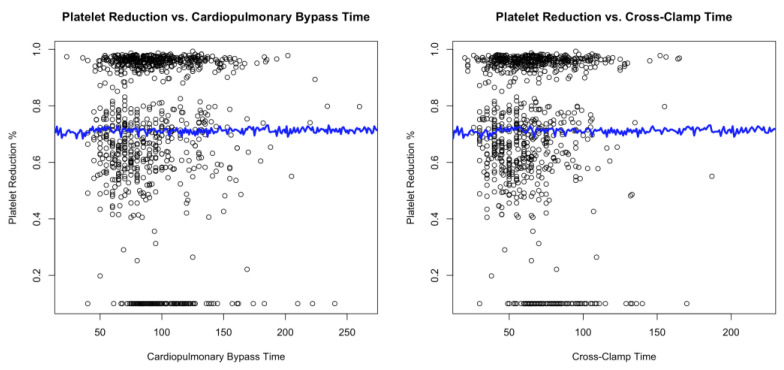
Correlation between platelet count variation and the cardiopulmonary and cross-clamp times.

A LOESS analysis showed that PR was not affected by CPB time (mean: −0.00, SE: 0.001, *p* = 0.635) or cross-clamp time (mean: −0.00, SE: 0.002, *p* = 0.051) in any group (Figure 2).

### 3.2. Secondary Outcomes

The total blood loss via drainage was greater in RDVs compared to the Stented (*p* < 0.001) or Stentless (*p* = 0.003) valves. Indeed, drainage blood loss was similar between the Stented and Stentless groups (*p* = 0.775).

Patients with RDV received more RBCs and FFPs than the other two groups (*p* < 0.001), as seen in Table 3. RDV and Stented patients required more platelet transfusions (*p* = 0.032) than Stentless patients, who did not receive any platelet transfusions.

Table 3 shows that bleeding problems were similar among the three groups (Stented: 8.1%, Stentless: 7.2%, RDV: 8.3%, overall *p* = 0.962). Re-thoracotomy for bleeding was performed more frequently in RDV patients (7.3%) compared to the Stented (4.9%) and Stentless (4.2%) groups (overall *p* = 0.313; Table 3).

Groups showed similar thrombotic and cardiovascular events (Table 3). For both the Stented and RDV groups, the median intensive care unit (ICU) and hospital length of stay (LoS) were similar (2 and 9 days, respectively). The Stentless group had a median ICU and Hospital LoS of 3 and 11 days, respectively; Table 3. In-hospital mortality rate was higher for the RDV (6%) compared to the other two groups (Stented: 2.3%; Stentless: 1.4%; *p* = 0.012).

**Figure 3:**
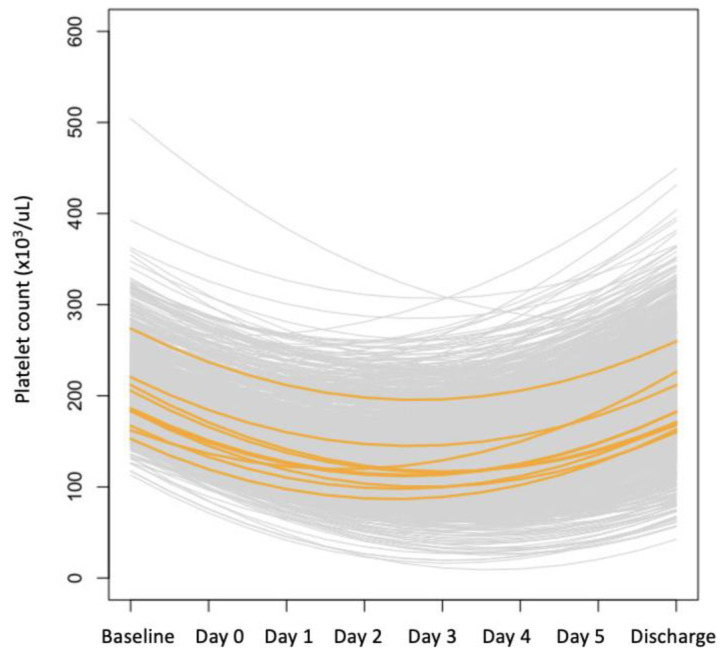
Predicted platelet variation over time in all patients (the orange lines represent random patients; they are graphically highlighted to better illustrate platelet variations over time).

**Figure 4:**
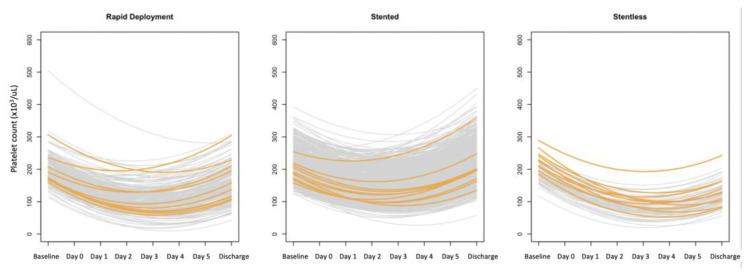
Predicted platelet variation over time in the three groups (the orange lines represent random patients; they are graphically highlighted to better illustrate platelet variations over time).

Using a mixed-model effect analysis of repeated measures, we obtained four coefficients: (1) pre-operative platelet count value (P1); (2) the coefficient of the platelet count variation (grad1); (3) the time to reach the minimum platelet count value (Tmin); and (4) the predicted minimum platelet count value (Pmin). The predicted platelet count variation over time is shown in Figure 3 and Figure 4. 

Multivariate regression analyses (Supplementary Materials) showed that in the overall population, the ischaemic strokes were significantly correlated with P1 (*p* = 0.026), grad1 (0.029), Tmin (*p* = 0.027), and Pmin (*p* = 0.033). The other outcomes were not significantly associated with platelet count variation. Analysing the subgroup types, bleeding events and in-hospital mortality were significantly associated with P1 (bleeding events, *p* = 0.028; in-hospital mortality, *p* = 0.036) and Pmin (bleeding events, *p* = 0.022; in-hospital mortality, *p* = 0.049) in the stented bioprostheses. Moreover, ischemic strokes were found to be associated with grad1 (*p* = 0.011) and Tmin (*p* = 0.010) in patients receiving stented bioprostheses. The other outcomes were not associated with platelet count variation. In the RDV, the drainage blood loss was significantly correlated with P1 (*p* = 0.013) and Pmin (*p* = 0.011) platelet count values. In the stentless patients, none of the considered secondary outcomes were significantly associated with the platelet count variation. After forcing PS weighting into all multivariate models in order to adjust the results, no changes were observed. 

## 4. Discussion

The current retrospective investigation demonstrated that regardless of the kind of bioprosthesis, peri-operative PR is frequent following the implantation of an aortic valve. A recent meta-analysis and comprehensive review [1] revealed similar findings. All bioprosthesis implantations were associated with a postoperative platelet count decrease, although the percentages varied: for stentless valves, the reduction ranged from 60% to 77%; for stented valves, it ranged from 35% to 55% [1]. The current study supports previous findings that patients who receive an RDV or a stentless valve are more likely to develop PR.

PR occurs early after an aortic bioprosthesis implantation (between the 2nd and the 3rd POD), as confirmed by our analysis, where the platelet count was even lower than 100,000/uL—especially in the RDV and Stentless groups. This phenomenon seems temporary, though; after the implantation of an RDV or a Stentless valve, the platelet count typically returns to normal within 7–10 days [1,2,3,4,5,6]. In the current study, a gradual increase in platelet count was also seen. Furthermore, compared to patients who had a stented prosthesis (27%), at the nadir, both RDV and Stentless patients had higher PR rates (57% and 56%, respectively). On the third POD, patients with stentless valves had a PR rate of almost 77%, according to Yerekaban and colleagues [2]. Other research [4,14,15] has reported similar findings. In small, retrospective, observational studies, other researchers have found a comparable variation in platelet count in individuals with RDV [3,6,16]. In a recent prospective randomised study [17], Lorusso and colleagues reported a greater PR for the RDV (46.6%) than for stented prostheses (32.5%).

Discussions over the abrupt drop in platelet count following the implantation of an RDV and a stentless prosthesis have been very heated [3,4,5,6,8,14,15,16]. This phenomenon was initially attributed to the CPB. Vogt and colleagues discovered a correlation between CPB and early post-operative PR in a retrospective observational study that examined thrombocytopenia following surgical and transcatheter bioprosthesis [7]. Despite the surgical cohort’s size (over 1000 patients), their study suffers from a significant selection bias, as the analysis included combined procedures that required longer CPB periods. This conclusion should therefore be interpreted with care. On the other hand, our findings indicated that CPB and cross-clamp times had no impact on PR. Additionally, these times were particularly low in RDV patients, i.e., those with the highest PR. As a result, we looked for the source of PR within the prosthesis itself.

Despite being mainly made by pericardium, the material of a tissue valve does not have perfect biocompatibility or hemocompatibility [18]. Furthermore, the prosthesis’ design could elicit mechanical platelet destruction [2,5,9]. In particular, the prosthesis’ size could play a pivotal role in the platelet count variation. Small valve sizes are usually thought to be the main cause of blood turbulence, resulting in platelet activation or destruction [3,14,19]. Mujtaba and associates discovered a higher PR in the smallest stented valves (48%) and RDVs (66%) [6]. On the other hand, we discovered that the platelet count varies between all three kinds of prostheses. Our study found a higher platelet reduction in patients with small stented prostheses, which is supported by the literature. Patients who received an RDV or a stentless valve, on the other hand, had higher platelet decreases with larger prosthesis sizes. A larger prosthesis may increase the possibility of blood components being exposed to a larger foreign surface, resulting in an increased inflammatory response and accompanying platelet activation. Furthermore, cardiac surgeons’ excessive oversizing during the initial years of implantations may have caused the PR to be even higher in the RDV. Oversizing, in fact, might cause a suboptimal expansion of the valve, resulting in high gradients and paravalvular leaks [20]. Hence, platelet dysfunction, rupture, and the shedding of receptors are natural consequences of a turbulent flow [2,5,9].

The literature is devoid of strong evidence concerning PR after aortic bioprosthesis implantation because it was often thought of as a drawback with no effect on patients’ clinical outcomes. Stegmeier and co-workers did not find any association between PR and mortality, blood loss, bleeding events, or the duration of hospitalisation [16]. Likewise, Lorusso and colleagues did not report any difference in blood loss, platelet and RBC transfusions, bleeding events, or strokes between RDV and stented prostheses [17]. Similarly, Repossini and associates did not report any bleeding or thromboembolic events after stentless implantations [21]. On the other hand, we discovered that in the entire enrolled group, platelet count fluctuation was associated with ischemic stroke. In particular, regardless of the type of prosthesis, ischemic strokes were discovered to be substantially linked with the platelet count decline, the minimal platelet count value, and the speed of the platelet count drop. The risk of stroke within 30 days among 67,292 patients after isolated SAVR has been reported to be as high as 1.5%, according to the Society of Thoracic Surgeons (STS) database [22]. In 6523 patients undergoing SAVR, the German Aortic Valve Registry found a 1.3% in-hospital stroke rate [23]. In the present study, the incidence of stroke in the stented bioprosthesis group was in line with the value found in the literature (1.7%). However, we found a higher rate of stroke in the RDV group; this was also the group that experienced higher PR. We could speculate that this finding could be the result of increasing platelet consumption in the process of their activation and subsequent thrombosis. Platelets have a pivotal role in thrombus formation that may initiate the symptoms of stroke. We could hypothesise that platelet reactivity is an additional risk factor for cerebrovascular events, similar to what Jimenez Diaz and colleagues reported for a population undergoing trans-catheter aortic valve implantation [24].

In addition, in our analysis, the majority of RDV patients had the worst postoperative clinical scenario: (1) a much higher drainage blood loss; (2) a high rate of RBCs and FFP transfusions; (3) more re-thoracotomies for bleeding; and (4) a higher in-hospital mortality rate. Additionally, to a lesser extent, stentless patients required more blood product transfusions. Despite what was previously reported, PR after bioprosthesis implantation does not seem completely risk-free [17]. Nevertheless, these results should be interpreted cautiously, given the limitations of the present study.

### Strengths and Limitations

The main strength of our study is its multicenter design and the large population of enrolled patients demonstrating platelet kinetics after surgical biological valve implantation. The main limitation of this study is that the stentless group represented a small cohort compared to the other two groups. A selection bias may have affected the outcomes due to this imbalance. Therefore, the results observed in this group should be taken with caution. Moreover, given the retrospective design of the study, some of the post-operative echocardiographic data (i.e., post-operative paravalvular leaks) were missing. Similarly, incomplete data for some variables (i.e., ICU LoS, in-hospital LoS) may skew some results. In addition, this study lacks information and analyses regarding the role of heparin-induced thrombocytopenia (HIT) as a possible cause of PR and lacks some relevant echocardiographic data such as the perioperative annular size or postoperative paravalvular leaks. However, we can assume that the overall outcomes were not biased because of the large population included and the very low incidence of HIT after heart surgery (0.3%) [25].

## 5. Conclusions

A considerable but temporary platelet drop is linked to the implantation of an aortic bioprosthesis. Patients who get an RDV or a stentless prosthesis are more likely to have a clinically significant PR and accompanying side effects. Platelet reactivity could also be an additional risk factor for post-operative stroke. Current explanations for this phenomenon remain speculative. Further prospective studies could try to explain the mechanism underlying the platelet reduction in relation to the prosthesis valve type and size.

## Figures and Tables

**Table 1:** Preoperative characteristics of the stented, rapid deployment, and stentless bioprostheses groups.

	Stented Bioprostheses (n = 944)	Rapid Deployment Bioprostheses (n = 218)	Stentless Bioprostheses (n = 71)	Overall *p* Value	*p* Value (Stented vs. RDV)	*p* Value (RDV vs. Stentless)	*p* Value (Stented vs. Stentless)
Age (years)	70.8 ± 9.3	72.6 ± 6.0	71.2 ± 7.3	0.022	0.015	0.450	0.323
Male (%)	551 (58.4)	118 (54.1)	49 (69.0)	<0.001	0.001	0.001	0.078
BMI	27.8 ± 4.8	28.7 ± 4.7	27.4 ± 4.2	0.044	0.065	0.113	0.671
BSA	1.80 ± 0.18	1.79 ± 0.20	1.82 ± 0.18	0.041	0.368	0.032	0.120
Hypertension (%)	706 (74.8)	164 (75.2)	41 (57.7)	0.006	0.892	0.005	0.002
Diabetes Mellitus (%)	214 (22.7)	73 (33.5)	23 (32.4)	0.001	0.001	0.865	0.062
NYHA class				0.004	0.025	0.027	0.003
I (%)	202 (21.4)	30 (13.8)	7 (9.9)				
II (%)	339 (41.3)	97 (44.5)	45 (63.4)				
III (%)	323 (34.2)	88 (40.4)	17 (23.9)				
IV (%)	29 (3.1)	3 (1.4)	2 (2.8)				
Smoking (%)	232 (24.6)	64 (29.4)	24 (33.8)	0.104	0.144	0.480	0.084
Dyslipidaemia (%)	404 (42.8)	142 (65.1)	15 (21.1)	<0.001	<0.001	<0.001	<0.001
Preop ASA (%)	394 (41.7)	73 (33.5)	41 (57.7)	0.001	0.025	<0.001	0.009
Preop LVEF	54.4 ± 8.8	56.2 ± 8.0	56.5 ± 11.3	0.005	0.015	0.966	0.118
Preop Mean Gradient	52.2 ± 17.0	56.2 ± 14.5	47.1 ± 14.9	<0.001	0.006	<0.001	0.040

Abbreviations: *RDV*, rapid deployment valve; *BMI*, body mass index; *BSA*, body surface area; *NYHA class*, New York Heart Association class; *Preop*, preoperatively; *ASA*, aspirin; *LVEF*, left ventricle ejection fraction.

**Table 2:** Post-operative platelet counts of the stented, rapid deployment, and stentless bioprostheses groups.

	Stented Bioprostheses (n = 944)	Rapid Deployment Bioprostheses (n = 218)	Stentless Bioprostheses (n = 71)	Overall *p* Value	*p* Value (Stented vs. RDV)	*p* Value (RDV vs. Stentless)	*p* Value (Stented vs. Stentless)
Pre-operatively PC	217 ± 65	218 ± 62	211 ± 53	0.700	0.981	0.691	0.705
Day 0 PC	161 ± 62	137 ± 51	123 ± 46	<0.001	<0.001	0.229	<0.001
Day 1 PC	157 ± 55	136 ± 50	118 ± 37	<0.001	<0.001	0.059	<0.001
Day 2 PC	146 ± 64	98 ± 42	104 ± 41	<0.001	<0.001	0.758	<0.001
Day 3 PC	158 ± 72	93 ± 42	92 ± 39	<0.001	<0.001	0.997	<0.001
Day 4 PC	175 ± 77	103 ± 51	89 ± 44	<0.001	<0.001	0.417	<0.001
Day 5 PC	190 ± 80	105 ± 53	93 ± 42	<0.001	<0.001	0.518	<0.001
PC at discharge	214 ± 82	170 ± 93	119 ± 42	<0.001	<0.001	<0.001	<0.001
Number of patients with PC <100,000/uL on Day 2	166 (18%)	118 (54%)	31 (44%)	<0.001	<0.001	0.946	<0.001
Number of patients with PC <100,000/uL on Day 3	136 (14%)	108 (50%)	39 (55%)	<0.001	<0.001	0.931	<0.001

**Abbreviations**: RDV, rapid deployment valve; PC, platelet count.

**Table 3:** Post-operative details of the stented, rapid deployment, and stentless bioprostheses groups.

	Stented Bioprostheses (n = 944)	Rapid Deployment Bioprostheses (n = 218)	Stentless Bioprostheses (n = 71)	Overall *p* Value	*p* Value (Stented vs. RDV)	*p* Value (RDV vs. Stentless)	*p* Value (Stented vs. Stentless)
Drainage blood loss (mL)	579 ± 533	782 ± 472	534 ± 237	<0.001	<0.001	0.003	0.775
RBC transfused	0.90 ± 1.30	1.88 ± 2.60	1.30 ± 1.34	<0.001	<0.001	0.001	0.006
FFP transfused	0.48 ± 1.21	1.25 ± 2.06	1.12 ± 1.51	<0.001	<0.001	0.545	<0.001
PLT transfused	0.10 ± 0.68	0.14 ± 0.60	0 ± 0	0.032	0.603	0.025	0.080
Bleeding	76 (8.1%)	18 (8.3%)	5 (7.2%)	0.962	0.989	0.940	0.881
Reoperation for bleeding	46 (4.9%)	16 (7.3%)	3 (4.2%)	0.313	0.144	0.358	1.000
Ischemic stroke	16 (1.7%)	5 (2.3%)	0 (0%)	0.431	0.572	0.339	0.269
TIA	10 (1%)	0 (0%)	0 (0%)	0.214	1.000	---	1.000
Intracranial bleeding	4 (0.4%)	0 (0%)	0 (0%)	0.541	1.000	---	1.000
Gastrointestinal bleeding	5 (0.5%)	2 (0.9%)	0 (0%)	0.637	0.622	1.000	1.000
ICU length of stay (days)	2 (0–81)	2 (1–90)	3 (0–21)	*	*	*	*
Hospital length of stay (days)	9 (0–81)	9 (0–114)	11 (6–24)	*	*	*	*
In-hospital mortality	22 (2.3%)	13 (6.0%)	1 (1.4%)	0.012	0.005	0.200	1.000
**Echocardiographic features**							
Post-op LVEF	54.6 ± 7.2	54.6 ± 5.1	54.6 ± 8.9	0.995	<0.001	<0.001	0.042
Post-op Mean gradient	13.5 ± 5.6	14.7 ± 8.8	11.3 ± 6.1	<0.001	0.012	0.735	0.224

Abbreviations: RDV, rapid deployment valve;RBC, red blood cell; FFP, fresh frozen plasma; PLT, platelet; TIA, transient ischemic attack; ICU, intensive care unit, LVEF, left ventricular ejection fraction. * because of missing data, the software is not able to provide a valid inference; therefore, no *p*-value was calculated.

## Data Availability

The data presented in this study are available on request from the corresponding author.

## Supplementary Materials

The following supporting information can be downloaded at: https://www.mdpi.com/article/10.3390/jcm12237414/s1.
