# Supplementary material for: Platelet Reduction after Aortic Bioprosthesis Implantation: Results from the PORTRAIT Study
Source: J Clin Med. 2023 Nov 29;12(23):7414. doi: 10.3390/jcm12237414 (PMC10707158; doi:10.3390/jcm12237414)
Supplement: Supplementary file 1 [file jcm-12-07414-s001.zip › jcm-2715071-supplementary.pdf]

Supplemental Material

Platelet Reduction after Aortic Bioprosthesis Implantation:

Results from the PORTRAIT Study

Supplemental Figures

Figure S1. Love plot reporting balancing among the three groups before (green triangles) and after (red circles) the weighting……………………………………..………………….... page 2

Supplemental Tables

Supplemental Table S1. Surgical details of the stented, rapid deployment, and stentless bioprostheses groups. …………………………………………………….....………………page 4

Tables adjusted by overlap weighting

Supplemental Table S2. Post-operative platelet count of the stented, rapid deployment, and stentless bioprostheses groups, adjusted by overlap weighting………………………………page 6

Supplemental Table S3. Surgical details of the stented, rapid deployment, and stentless bioprostheses groups, adjusted by overlap weighting.………………………………………page 7

Supplemental Table S4. Post-operative platelet count of the stented, rapid deployment, and stentless bioprostheses groups, adjusted by overlap weighting.……………………………page 8

Supplemental Table S5. Post-operative details of the stented, rapid deployment, and stentless bioprostheses groups, adjusted by overlap weighting ………………………………………page 9

Statistical Analysis

Multivariate analysis in all patients………………………………………………………..page 10

Multivariate analysis in stented patients…………………………………………………..page 14

Multivariate analysis in rapid deployment patients……………………………………….page 18

Multivariate analysis in stentless patients……………………………………...……..……page 21

Figure S1. Love plot reporting balancing among the three groups before (green triangles) and after (red circles) the weighting.

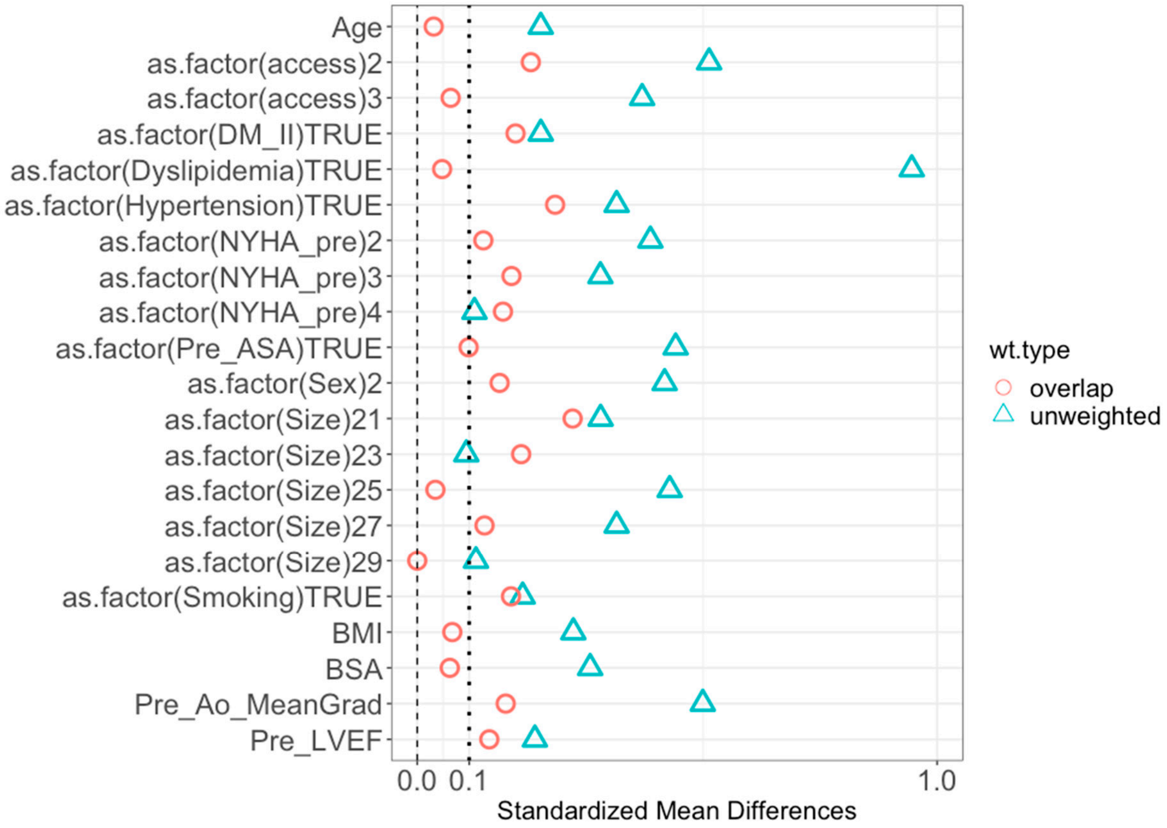

Tables

Supplemental Table S1. Surgical details of the stented, rapid deployment, and stentless bioprostheses groups.

	Stented bioprostheses (n=944)	Rapid deployment bioprostheses (n=218)	Stentless bioprostheses (n=71)	Overall p value	P value (Stented vs. RDV)	P value (RDV vs Stentless)	P value (Stented vs Stentless)
Access				< 0.001	< 0.001	< 0.001	0.010
Full sternotomy (%)	589 (62.4%)	100 (45.9%)	57 (80.3%)				
Mini sternotomy (%)	340 (36.0%)	96 (44.0%)	13 (18.3%)				
Right thoracotomy (%)	15 (1.6%)	22 (10.1%)	1 (1.4%)				
**Prosthesis size**				< 0.001	< 0.001	0.012	<0.001
**19mm**	98 (10.4)	7 (3.2)	5 (7.0)				
**21mm**	294 (31.1)	35 (16.1)	19 (26.8)				
**23mm**	351 (37.2)	76 (34.9)	28 (39.4)				
**25mm**	179 (19.0)	74 (33.9)	10 (14.1)				
**27mm**	18 (1.9)	26 (11.9)	9 (12. 7)				
**29mm**	4 (0.4)	0 (0)	0 (0)				
CPB time (min)	99.5±30.1	76.4±25.4	106.5±28.8	< 0.001	< 0.001	<0.001	0.133
Cross-clamp time (min)	22.3±7.61	48.9±18.7	85.6±24.0	< 0.001	< 0.001	< 0.001	< 0.001

Abbreviations: *RDV*, rapid deployment valve; *CPB*, cardiopulmonary bypass.

Tables adjusted by overlap weighting

Supplemental Table S2. Post-operative platelet count of the stented, rapid deployment, and stentless bioprostheses groups, adjusted by overlap weighting.

	Stented bioprostheses (n=944)	Rapid deployment bioprostheses (n=218)	Stentless bioprostheses (n=71)	Overall p value	P value (Stented vs. RDV)	P value (RDV vs Stentless)	P value (Stented vs Stentless)
Pre-operatively PC	218±65	219±62	216±53	0.910	0.792	0.666	0.867
Day 0 PC	167±62	142±52	126±47	< 0.001	< 0.001	< 0.001	< 0.001
Day 1 PC	161±55	137±50	120±38	< 0.001	< 0.001	< 0.001	< 0.001
Day 2 PC	169±64	99±42	106±41	< 0.001	< 0.001	0.189	< 0.001
Day 3 PC	159±72	96±43	97±40	< 0.001	< 0.001	0.994	< 0.001
Day 4 PC	189±77	106±52	94±45	< 0.001	< 0.001	0.064	< 0.001
Day 5 PC	210±80	100±53	100±43	< 0.001	< 0.001	0.999	< 0.001
PC at discharge	215±82	164±93	126±42	< 0.001	< 0.001	< 0.001	< 0.001
Number of patients with PC <100.000/uL on Day 2	166 (18%)	118 (54%)	31 (44%)	< 0.001	< 0.001	0.464	< 0.001
Number of patients with PC <100.000/uL on Day 3	136 (14%)	108 (50%)	39 (55%)	< 0.001	< 0.001	0.127	< 0.001

Abbreviations: *RDV*, rapid deployment valve; *PC*, platelet count

Supplemental Table S3. Surgical details of the stented, rapid deployment, and stentless bioprostheses groups, adjusted by overlap weighting.

	Stented bioprostheses (n=944)	Rapid deployment bioprostheses (n=218)	Stentless bioprostheses (n=71)	Overall p value	P value (Stented vs. RDV)	P value (RDV vs Stentless)	P value (Stented vs Stentless)
Access				< 0.001	< 0.001	< 0.001	0.020
Full sternotomy (%)	589 (62.4%)	100 (45.9%)	57 (80.3%)				
Mini sternotomy (%)	340 (36.0%)	96 (44.0%)	13 (18.3%)				
Right thoracotomy (%)	15 (1.6%)	22 (10.1%)	1 (1.4%)				
**Prosthesis size**				< 0.001	< 0.001	0.012	<0.001
**19mm**	98 (10.4)	7 (3.2)	5 (7.0)				
**21mm**	294 (31.1)	35 (16.1)	19 (26.8)				
**23mm**	351 (37.2)	76 (34.9)	28 (39.4)				
**25mm**	179 (19.0)	74 (33.9)	10 (14.1)				
**27mm**	18 (1.9)	26 (11.9)	9 (12. 7)				
**29mm**	4 (0.4)	0 (0)	0 (0)				
CPB time (min)	99.5±30.1	76.4±25.4	106.5±28.8	< 0.001	< 0.001	<0.001	0.167
Cross-clamp time (min)	7.61±22.3	48.9±18.7	85.6±24.0	< 0.001	< 0.001	< 0.001	< 0.001

Abbreviations: *RDV*, rapid deployment valve; *CPB*, cardiopulmonary bypass.

Supplemental Table S4. Post-operative platelet count of the stented, rapid deployment, and stentless bioprostheses groups, adjusted by overlap weighting.

	**Stented bioprostheses (n=944)**	**Rapid deployment bioprostheses (n=218)**	**Stentless bioprostheses (n=71)**	**Overall p value**	**P value (Stented vs. RDV)**	**P value (RDV vs Stentless)**	**P value (Stented vs Stentless)**
Pre-operatively PC	218±65	219±62	216±53	0.910	0.792	0.666	0.867
Day 0 PC	167±62	142±52	126±47	< 0.001	< 0.001	< 0.001	< 0.001
Day 1 PC	161±55	137±50	120±38	< 0.001	< 0.001	< 0.001	< 0.001
Day 2 PC	159±64	99±42	106±41	< 0.001	< 0.001	0.189	< 0.001
Day 3 PC	169±72	96±43	97±40	< 0.001	< 0.001	0.994	< 0.001
Day 4 PC	189±77	106±52	94±45	< 0.001	< 0.001	0.064	< 0.001
Day 5 PC	210±80	100±53	100±43	< 0.001	< 0.001	0.999	< 0.001
PC at discharge	219±82	164±93	126±42	< 0.001	< 0.001	< 0.001	< 0.001
Number of patients with PC <100.000/uL on Day 2	166 (18%)	118 (54%)	31 (44%)	< 0.001	< 0.001	0.464	< 0.001
Number of patients with PC <100.000/uL on Day 3	136 (14%)	108 (50%)	39 (55%)	< 0.001	< 0.001	0.127	< 0.001

Abbreviations: *RDV*, rapid deployment valve; *PC*, platelet count.

Supplemental Table S5. Post-operative details of the stented, rapid deployment, and stentless bioprostheses groups, , adjusted by overlap weighting

	Stented bioprostheses (n=944)	Rapid deployment bioprostheses (n=218)	Stentless bioprostheses (n=71)	Overall p value	P value (Stented vs. RDV)	P value (RDV vs Stentless)	P value (Stented vs Stentless)
Drainage blood loss (ml)	583±534	761±471	584±238	<0.001	<0.001	<0.001	0.999
RBC transfused	0.79±1.29	1.98±2.50	1.29±1.34	< 0.001	< 0.001	<0.001	0.002
FFP transfused	0.39±1.26	0.91±2.06	1.12±1.51	< 0.001	< 0.001	0.085	<0.001
PLT transfused	0.09±0.69	0.20±0.60	0±0	<0.001	0.036	<0.001	0.108
Bleeding	76 (8.1%)	18 (8.3%)	5 (7.2%)	0.442	0.314	0.206	0.661
Reoperation for bleeding	46 (4.9%)	16 (7.3%)	3 (4.2%)	0.313	0.149	0.349	0.800
Ischemic stroke	16 (1.7%)	5 (2.3%)	0 (0%)	0.949	0.746	0.997	0.997
TIA	10 (1%)	0 (0%)	0 (0%)	1.000	0.993	1.000	0.996
Intracranial bleeding	4 (0.4%)	0 (0%)	0 (0%)	1.000	0.995	1.000	0.997
Gastrointestinal bleeding	5 (0.5%)	3 (1.3%)	0 (0%)	0.965	0.789	0.997	0.997
ICU length of stay (days)	2 (0-81)	2 (1-90)	3 (0-21)	*	*	*	*
Hospital length of stay (days)	9 (0-81)	9 (0-114)	11 (6-24)	*	*	*	*
In-hospital mortality	22 (2.3%)	13 (6.0%)	1 (1.4%)	0.006	0.003	0.120	0.659

Multivariate Analysis - Overall patients

Drainage blood loss

Coefficients

Model		Unstandardized	Standard Error	Standardized	t	p	95% CI Lower	95% CI Upper
H_0	(Intercept)	606.450	16.081		37.713	< .001	574.896	638.005
H_1	(Intercept)	2339.578	5590.947		0.418	0.676	-8631.394	13310.550
	P1	0.540	3.586	0.044	0.150	0.880	-6.498	7.577
	grad1	-152.716	481.739	-2.274	-0.317	0.751	-1098.020	792.588
	tmin	-1774.388	5600.052	-2.274	-0.317	0.751	- 12763.226	9214.449
	Pmin	-0.610	3.615	-0.055	-0.169	0.866	-7.703	6.484

Bleeding events

Coefficients

	Estimate	Standard Error	Odds Ratio	z	Wald Test: Wald Statistic	Wald Test: df	Wald Test: p	95% Confidence interval: Lower bound	95% Confidence interval: Upper bound
(Intercept)	5.994	36.449	400.877	0.164	0.027	1	0.869	-65.445	77.432
P1	0.019	0.022	1.019	0.854	0.730	1	0.393	-0.025	0.063
grad1	-0.543	3.147	0.581	- 0.173	0.030	1	0.863	-6.710	5.625
tmin	-6.999	36.556	9.131e -4	- 0.191	0.037	1	0.848	-78.648	64.650
Pmin	-0.023	0.023	0.978	- 1.003	1.005	1	0.316	-0.067	0.022

Note. Bleeding level '1' coded as class 1.

Re-operation for bleeding

Coefficients

					Wald Test			95% Confidence interval	
	**Estimate**	**Standard Error**	**Odds Ratio**	**z**	**Wald Statistic**	**df**	**p**	**Lower bound**	**Upper bound**
(Intercept)	10.778	44.444	47939.131	0.242	0.059	1	0.808	-76.331	97.887
P1	0.014	0.027	1.015	0.534	0.285	1	0.593	-0.039	0.068
grad1	-0.968	3.836	0.380	-0.252	0.064	1	0.801	-8.487	6.552
tmin	-11.885	44.572	6.892e -6	-0.267	0.071	1	0.790	-99.246	75.475
Pmin	-0.019	0.027	0.981	-0.698	0.487	1	0.485	-0.073	0.035

Note. Reop_bleeding level '1' coded as class 1.

Ischemic stroke

Coefficients

					Wald Test			95% Confidence interval	
	**Estimate**	**Standard Error**	**Odds Ratio**	**z**	**Wald Statistic**	**df**	**p**	**Lower bound**	**Upper bound**
(Intercept)	182.508	82.408	1.829e +79	2.215	4.905	1	0.027	20.991	344.026
P1	0.062	0.028	1.064	2.230	4.971	1	0.026	0.007	0.116
grad1	-15.490	7.115	1.875e -7	-2.177	4.739	1	0.029	-29.436	-1.544
tmin	-182.709	82.735	4.471e -80	-2.208	4.877	1	0.027	-344.868	-20.551
Pmin	-0.061	0.029	0.941	-2.134	4.554	1	0.033	-0.117	-0.005

Note. Stroke level '1' coded as class 1.

TIA

Coefficients

					Wald Test			95% Confidence interval	
	**Estimate**	**Standard Error**	**Odds Ratio**	**z**	**Wald Statistic**	**df**	**p**	**Lower bound**	**Upper bound**
(Intercept)	111.730	116.997	3.340e +48	0.955	0.912	1	0.340	-117.579	341.039
P1	-0.288	0.222	0.750	-1.296	1.680	1	0.195	-0.724	0.148
grad1	-12.585	10.041	3.422e -6	-1.253	1.571	1	0.210	-32.265	7.095
tmin	-136.115	116.583	7.693e -60	-1.168	1.363	1	0.243	-364.613	92.384
Pmin	0.314	0.223	1.369	1.405	1.973	1	0.160	-0.124	0.752

Note. TIA level '1' coded as class 1.

Intracranial bleeding

Coefficients

					Wald Test			95% Confidence interval	
	**Estimate**	**Standard Error**	**Odds Ratio**	**z**	**Wald Statistic**	**df**	**p**	**Lower bound**	**Upper bound**
(Intercept)	-285.314	269.397	1.230e -124	-1.059	1.122	1	0.290	-813.323	242.696
P1	-6.555	4.357	0.001	-1.505	2.264	1	0.132	-15.094	1.983
grad1	-18.794	20.360	6.884e -9	-0.923	0.852	1	0.356	-58.698	21.110
tmin	-9.350	189.162	8.693e -5	-0.049	0.002	1	0.961	-380.101	361.401
Pmin	6.584	4.361	723.320	1.510	2.279	1	0.131	-1.963	15.131

Note. Intra_cranial_haemorrhage level '1' coded as class 1.

Gastrointestinal bleeding

Coefficients

					Wald Test			95% Confidence interval	
	**Estimate**	**Standard Error**	**Odds Ratio**	**z**	**Wald Statistic**	**df**	**p**	**Lower bound**	**Upper bound**
(Intercept)	-64.635	137.536	8.498e -29	-0.470	0.221	1	0.638	-334.201	204.931
P1	0.014	0.092	1.014	0.146	0.021	1	0.884	-0.167	0.194
grad1	4.757	11.950	116.347	0.398	0.158	1	0.691	-18.665	28.178
tmin	56.002	138.412	2.096e +24	0.405	0.164	1	0.686	-215.280	327.284
Pmin	-0.016	0.096	0.984	-0.167	0.028	1	0.868	-0.204	0.172

Note. GI_bleeding level '1' coded as class 1.

In-hospital mortality

Coefficients

					Wald Test			95% Confidence interval	
	**Estimate**	**Standard Error**	**Odds Ratio**	**z**	**Wald Statistic**	**df**	**p**	**Lower bound**	**Upper bound**
(Intercept)	-41.387	60.820	1.061e -18	-0.680	0.463	1	0.496	-160.592	77.817
P1	0.049	0.033	1.050	1.478	2.183	1	0.140	-0.016	0.113
grad1	3.439	5.263	31.151	0.653	0.427	1	0.514	-6.877	13.754
tmin	38.732	61.080	6.622e +16	0.634	0.402	1	0.526	-80.982	158.446
Pmin	-0.050	0.034	0.951	-1.463	2.140	1	0.144	-0.117	0.017

Note. In_hospital_mortality level '1' coded as class 1.

Multivariate Analysis - Stented bioprostheses

Drainage blood loss

Coefficients

Model		Unstandardized	Standard Error	Standardized	t	p	95% CI Lower	95% CI Upper
H_0	(Intercept)	578.705	18.697		30.952	< .001	542.005	615.405
H_1	(Intercept)	933.986	6489.064		0.144	0.886	-11803.354	13671.327
	P1	2.642	4.817	0.208	0.548	0.584	-6.814	12.097
	grad1	-22.692	558.670	-0.289	-0.041	0.968	-1119.301	1073.918
	tmin	-338.361	6496.499	-0.371	-0.052	0.958	-13090.296	12413.573
	Pmin	-2.271	4.876	-0.180	-0.466	0.641	-11.842	7.300

Bleeding events

Coefficients

	Estimate	Standard Error	Odds Ratio	z	Wald Test: Wald Statistic	Wald Test: df	Wald Test: p	95% Confidence interval: Lower bound	95% Confidence interval: Upper bound
(Intercept)	-5.928	41.700	0.003	-0.142	0.020	1	0.887	-87.659	75.804
P1	0.054	0.025	1.055	2.198	4.830	1	0.028	0.006	0.102
grad1	0.684	3.600	1.981	0.190	0.036	1	0.849	-6.372	7.739
tmin	6.192	41.834	488.812	0.148	0.022	1	0.882	-75.800	88.184
Pmin	-0.057	0.025	0.944	-2.288	5.233	1	0.022	-0.107	-0.008

Note. Bleeding level '1' coded as class 1.

Re-operation for bleeding

Coefficients

					Wald Test			95% Confidence interval	
	Estimate	Standard Error	Odds Ratio	z	Wald Statistic	df	p	Lower bound	Upper bound
(Intercept)	12.590	52.183	293658.331	0.241	0.058	1	0.809	-89.687	114.868
P1	0.034	0.030	1.035	1.155	1.334	1	0.248	-0.024	0.092
grad1	-1.096	4.505	0.334	-0.243	0.059	1	0.808	-9.925	7.733
tmin	-13.964	52.348	8.617e-7	-0.267	0.071	1	0.790	-116.564	88.636
Pmin	-0.033	0.030	0.968	-1.097	1.203	1	0.273	-0.092	0.026

Note. Reop_bleeding level '1' coded as class 1.

Ischaemic stroke

Coefficients

					Wald Test			95% Confidence interval	
	Estimate	Standard Error	Odds Ratio	z	Wald Statistic	df	p	Lower bound	Upper bound
(Intercept)	261.435	101.969	3.467e+113	2.564	6.573	1	0.010	61.580	461.291
P1	0.063	0.035	1.065	1.828	3.340	1	0.068	-0.005	0.131
grad1	-22.416	8.805	1.840e-10	-2.546	6.481	1	0.011	-39.674	-5.158
tmin	-263.163	102.407	5.126e-115	-2.570	6.604	1	0.010	-463.876	-62.450
Pmin	-0.058	0.035	0.944	-1.647	2.711	1	0.100	-0.127	0.011

Note. Stroke level '1' coded as class 1.

TIA

Coefficients

					Wald Test			95% Confidence interval	
	**Estimate**	**Standard Error**	**Odds Ratio**	**z**	**Wald Statistic**	**df**	**p**	**Lower bound**	**Upper bound**
(Intercept)	115.538	116.892	1.504e +50	0.988	0.977	1	0.323	-113.566	344.641
P1	-0.274	0.222	0.760	-1.234	1.522	1	0.217	-0.709	0.161
grad1	-12.804	10.034	2.749e -6	-1.276	1.628	1	0.202	-32.470	6.862
tmin	-139.139	116.494	3.739e -61	-1.194	1.427	1	0.232	-367.464	89.186
Pmin	0.299	0.223	1.348	1.340	1.795	1	0.180	-0.138	0.736

Note. TIA level '1' coded as class 1.

Intracranial bleeding

Coefficients

					Wald Test			95% Confidence interval	
	**Estimate**	**Standard Error**	**Odds Ratio**	**z**	**Wald Statistic**	**df**	**p**	**Lower bound**	**Upper bound**
(Intercept)	-273.425	266.241	1.791e -119	-1.027	1.055	1	0.304	-795.247	248.397
P1	-6.729	4.423	0.001	-1.521	2.314	1	0.128	-15.398	1.941
grad1	-20.845	20.769	8.852e -10	-1.004	1.007	1	0.316	-61.552	19.862
tmin	-27.963	188.997	7.173e -13	-0.148	0.022	1	0.882	-398.391	342.464
Pmin	6.757	4.428	860.324	1.526	2.329	1	0.127	-1.921	15.436

Note. Intra_cranial_haemorrhage level '1' coded as class 1.

Gastrointestinal bleeding

Coefficients

					Wald Test			95% Confidence interval	
	**Estimate**	**Standard Error**	**Odds Ratio**	**z**	**Wald Statistic**	**df**	**p**	**Lower bound**	**Upper bound**
(Intercept)	-98.593	165.068	1.520e -43	-0.597	0.357	1	0.550	-422.119	224.934
P1	0.038	0.097	1.039	0.395	0.156	1	0.693	-0.152	0.229
grad1	7.935	14.451	2793.016	0.549	0.301	1	0.583	-20.389	36.258
tmin	92.134	167.051	1.031e +40	0.552	0.304	1	0.581	-235.280	419.548
Pmin	-0.048	0.100	0.953	-0.481	0.231	1	0.631	-0.244	0.148

Note. GI_bleeding level '1' coded as class 1.

In-hospital mortality

Coefficients

					Wald Test			95% Confidence interval	
	**Estimate**	**Standard Error**	**Odds Ratio**	**z**	**Wald Statistic**	**df**	**p**	**Lower bound**	**Upper bound**
(Intercept)	-81.562	76.970	3.784e -36	-1.060	1.123	1	0.289	-232.421	69.297
P1	0.074	0.035	1.077	2.102	4.420	1	0.036	0.005	0.143
grad1	7.057	6.658	1161.369	1.060	1.124	1	0.289	-5.992	20.107
tmin	79.854	77.315	4.787e +34	1.033	1.067	1	0.302	-71.682	231.389
Pmin	-0.071	0.036	0.932	-1.971	3.885	1	0.049	-0.141	-0.000

Note. In_hospital_mortality level '1' coded as class 1.

Multivariate Analysis - RDV

Drainage blood loss

Coefficients

							95% CI	
**Model**		**Unstandardized**	**Standard Error**	**Standardized**	**t**	**p**	**Lower**	**Upper**
H_0	(Intercept)	782.922	37.938		20.637	< .001	707.971	857.873
H_1	(Intercept)	21621.267	12782.690		1.691	0.093	-3637.496	46880.030
	P1	33.080	13.091	2.934	2.527	0.013	7.211	58.948
	grad1	-1230.619	1098.546	-15.990	-1.120	0.264	-3401.361	940.124
	tmin	-16198.154	12747.047	-18.112	-1.271	0.206	-41386.487	8990.180
	Pmin	-35.146	13.580	-3.114	-2.588	0.011	-61.980	-8.313

Bleeding events

Coefficients

					Wald Test			95% Confidence interval	
	**Estimate**	**Standard Error**	**Odds Ratio**	**z**	**Wald Statistic**	**df**	**p**	**Lower bound**	**Upper bound**
(Intercept)	8.407	84.434	4476.132	0.100	0.010	1	0.921	-157.082	173.895
P1	-0.140	0.141	0.869	-0.995	0.991	1	0.320	-0.417	0.136
grad1	-1.774	7.297	0.170	-0.243	0.059	1	0.808	-16.075	12.527
tmin	-15.748	84.298	1.448e -7	-0.187	0.035	1	0.852	-180.968	149.472
Pmin	0.126	0.141	1.134	0.889	0.790	1	0.374	-0.151	0.403

Note. Bleeding level '1' coded as class 1.

Re-operation for bleeding

Coefficients

					Wald Test			95% Confidence interval	
	**Estimate**	**Standard Error**	**Odds Ratio**	**z**	**Wald Statistic**	**df**	**p**	**Lower bound**	**Upper bound**
(Intercept)	17.938	95.030	6.171e +7	0.189	0.036	1	0.850	-168.317	204.193
P1	-0.165	0.188	0.848	-0.876	0.768	1	0.381	-0.533	0.204
grad1	-2.353	8.211	0.095	-0.287	0.082	1	0.774	-18.446	13.740
tmin	-22.293	94.565	2.080e -10	-0.236	0.056	1	0.814	-207.637	163.050
Pmin	0.137	0.188	1.147	0.729	0.532	1	0.466	-0.232	0.506

Note. Reop_bleeding level '1' coded as class 1.

Ischaemic stroke

Coefficients

					Wald Test			95% Confidence interval	
	**Estimate**	**Standard Error**	**Odds Ratio**	**z**	**Wald Statistic**	**df**	**p**	**Lower bound**	**Upper bound**
(Intercept)	14.775	174.295	2.609e +6	0.085	0.007	1	0.932	-326.837	356.386
P1	0.205	0.112	1.228	1.830	3.351	1	0.067	-0.015	0.425
grad1	1.270	14.897	3.561	0.085	0.007	1	0.932	-27.928	30.468
tmin	4.631	173.497	102.653	0.027	7.126e -4	1	0.979	-335.416	344.679
Pmin	-0.230	0.119	0.795	-1.922	3.695	1	0.055	-0.464	0.005

Note. Stroke level '1' coded as class 1.

TIA: Analysis not performed because of 0 events.

Intracranial bleeding: Analysis not performed because of 0 events.

Gastrointestinal bleeding

Coefficients

					Wald Test			95% Confidence interval	
	**Estimate**	**Standard Error**	**Odds Ratio**	**z**	**Wald Statistic**	**df**	**p**	**Lower bound**	**Upper bound**
(Intercept)	-3473.747	3598.809	0.000	-0.965	0.932	1	0.334	-10527.283	3579.789
P1	-32.496	33.659	7.712e-15	-0.965	0.932	1	0.334	-98.466	33.474
grad1	-142.347	149.077	1.512e-62	-0.955	0.912	1	0.340	-434.533	149.839
tmin	-28.367	254.124	4.788e-13	-0.112	0.012	1	0.911	-526.442	469.707
Pmin	32.501	33.658	1.303e+14	0.966	0.932	1	0.334	-33.467	98.469

Note. GI_bleeding level '1' coded as class 1.

In-hospital mortality

Coefficients

					Wald Test			95% Confidence interval	
	**Estimate**	**Standard Error**	**Odds Ratio**	**z**	**Wald Statistic**	**df**	**p**	**Lower bound**	**Upper bound**
(Intercept)	82.805	109.032	9.154e+35	0.759	0.577	1	0.448	-130.894	296.503
P1	-0.069	0.140	0.934	-0.489	0.239	1	0.625	-0.344	0.206
grad1	-9.005	9.216	1.227e-4	-0.977	0.955	1	0.328	-27.068	9.057
tmin	-99.637	107.241	5.348e-44	-0.929	0.863	1	0.353	-309.826	110.552
Pmin	0.064	0.145	1.066	0.444	0.197	1	0.657	-0.220	0.349

Note. In_hospital_mortality level '1' coded as class 1.

Multivariate Analysis - Stentless bioprostheses

Drainage blood loss

Coefficients

							95% CI	
**Model**		**Unstandardized**	**Standard Error**	**Standardized**	**t**	**p**	**Lower**	**Upper**
H_0	(Intercept)	534.444	30.032		17.796	< .001	474.411	594.478
H_1	(Intercept)	-9226.573	11012.053		-0.838	0.406	-31269.591	12816.445
	P1	-33.928	26.302	-4.492	-1.290	0.202	-86.578	18.721
	grad1	362.050	964.296	6.477	0.375	0.709	-1568.198	2292.298
	tmin	5957.200	10956.541	9.161	0.544	0.589	-15974.699	27889.099
	Pmin	33.603	26.575	4.410	1.264	0.211	-19.593	86.799

Bleeding events

Coefficients

					Wald Test			95% Confidence interval	
	**Estimate**	**Standard Error**	**Odds Ratio**	**z**	**Wald Statistic**	**df**	**p**	**Lower bound**	**Upper bound**
(Intercept)	135.673	219.494	8.353e+58	0.618	0.382	1	0.536	-294.528	565.873
P1	-0.728	0.972	0.483	-0.749	0.560	1	0.454	-2.633	1.178
grad1	-23.713	18.473	5.031e -11	-1.284	1.648	1	0.199	-59.919	12.494
tmin	-234.722	200.524	1.152e-102	-1.171	1.370	1	0.242	-627.741	158.297
Pmin	0.692	0.974	1.998	0.710	0.505	1	0.478	-1.218	2.602

Note. Bleeding level '1' coded as class 1.

Re-operation for bleeding

Coefficients

	Estimate	Standard Error	Odds Ratio	z	Wald Test			95% Confidence interval	
					Wald Statistic	df	p	Lower bound	Upper bound
(Intercept)	-126.297	303.674	1.412e -55	-0.416	0.173	1	0.677	-721.487	468.893
P1	-1.463	1.616	0.231	-0.905	0.819	1	0.365	-4.632	1.705
grad1	-14.107	22.018	7.471e -7	-0.641	0.411	1	0.522	-57.261	29.047
tmin	-79.055	233.596	4.644e -35	-0.338	0.115	1	0.735	-536.895	378.785
Pmin	1.326	1.594	3.767	0.832	0.693	1	0.405	-1.797	4.450

Note. Reop_bleeding level '1' coded as class 1.

Ischaemic stroke: Analysis not performed because of 0 events.

TIA: Analysis not performed because of 0 events.

Intracranial bleeding: Analysis not performed because of 0 events.

Gastrointestinal bleeding: Analysis not performed because of 0 events.

In-hospital mortality

Coefficients

	Estimate	Standard Error	Odds Ratio	z	Wald Test			95% Confidence interval	
					Wald Statistic	df	p	Lower bound	Upper bound
(Intercept)	-3303.044	15657664.603	0.000	-2.110e -4	4.450e -8	1	1.000	-30691761.748	30685155.659
P1	-6.284	77278.987	0.002	-8.131e -5	6.612e -9	1	1.000	-151470.315	151457.747
grad1	232.277	1076037.827	7.524e +100	2.159e -4	4.660e -8	1	1.000	-2108763.109	2109227.663
tmin	2945.445	12443006.890	∞	2.367e -4	5.603e -8	1	1.000	-24384899.918	24390790.809
Pmin	5.446	78548.658	231.750	6.933e -5	4.806e -9	1	1.000	-153947.095	153957.986

Note. In_hospital_mortality level '1' coded as class 1.
